# Mitigation of 3.5 GHz Electromagnetic Field-Induced BV2 Microglial Cytotoxicity by Polydeoxyribonucleotide

**DOI:** 10.3390/cimb47060386

**Published:** 2025-05-22

**Authors:** Shailashree Pachhapure, Amila Mufida, Qun Wei, Jong-Soon Choi, Byeong-Churl Jang

**Affiliations:** 1 Department of Molecular Medicine, College of Medicine, Keimyung University, Daegu 42601, Republic of Korea; shailashree@kmu.kr (S.P.); amilamufida@naver.com (A.M.); 2 Department of Medical Engineering, College of Engineering, Keimyung University, Daegu 42601, Republic of Korea; weiqun@kmu.ac.kr; 3 Biological Disaster Analysis Group, Division of Convergence Biotechnology, Korea Basic Science Institute, Daejeon 34126, Republic of Korea; 4 College of Medicine, Chung-Ang University, Seoul 06974, Republic of Korea

**Keywords:** electromagnetic field (EMF), 3.5 GHz, polydeoxyribonucleotide (PDRN), BV2 microglial cells, oxidative stress (ROS), SAPKs, apoptosis, A2A receptor

## Abstract

Emerging evidence highlights the biological risks associated with electromagnetic fields (EMFs) generated by electronic devices. The toxic effects and mechanisms induced by exposure to EMFs on microglial cells and natural substances that inhibit them are limited to date. Here, we investigated whether exposure to 3.5 GHz EMF radiation, potentially generated by smartphones working in 5G communication or cooking using microwave ovens, affects the growth of BV2 mouse microglial cells and polydeoxyribonucleotide (PDRN), a DNA preparation derived from salmon sperm, inhibits it. Of note, exposure to 3.5 GHz EMF radiation for 2 h markedly inhibited the growth and triggered apoptosis in BV2 cells, characterized by the reduced number of surviving cells, increased genomic DNA fragmentation, increased reactive oxygen species (ROS) levels, and altered phosphorylation and expression levels of JNK-1/2, p38 MAPK, ERK-1/2, eIF-2α, and procaspase-9. Pharmacological inhibition studies revealed that JNK-1/2 and p38 MAPK activation and ROS generation were crucial for 3.5 GHz EMF-induced BV2 cytotoxicity. Of interest, PDRN effectively countered these effects by inhibiting the activation of JNK-1/2, p38 MAPK, and caspase-9, and the production of ROS, although it did not affect eIF-2 phosphorylation. In conclusion, this study is the first to report that PDRN protects against 3.5 GHz EMF-induced toxicities in BV2 microglial cells, and PDRN’s protective effects on 3.5 GHz EMF-induced BV2 cytotoxicity are mediated primarily by modulating ROS, JNK-1/2, p38 MAPK, and caspase-9.

## 1. Introduction

Electromagnetic fields (EMFs) at high frequencies, ranging from 2 kHz to 300 GHz, are non-ionizing radiation emitted by various electronic devices and wireless communication systems, such as smartphones, Wi-Fi routers, and microwave ovens. The widespread use of these devices and technologies has raised public concerns about potential health risks, particularly acute or chronic EMF exposure [1,2]. In particular, the brain, due to its complex electromagnetic and electrophysiological activities, is especially vulnerable to EMF exposure, which can penetrate the skull and potentially induce neurotoxicity [3,4,5].

Research has shown that EMF exposure at specific frequencies can affect brain tissue and neuronal function. For instance, it has been shown that exposure to EMFs at 835 MHz for 8 h daily for 3 months induces neurotoxicity in the human cerebral cortex and cultured cortical neurons, as well as the alteration of glycine receptors for neurotransmitters in the auditory brainstem of mice [6]. Furthermore, long-term use of mobile phones at 1.9 GHz is associated with increased brain tumor risks [7], and exposure to 1.8 GHz EMFs for 48 h can disrupt neurite outgrowth [8]. Of further note, several in vitro and in vivo studies have indicated potential mechanisms and factors associated with EMF-induced neurotoxicity, including the phosphorylation (activation) of stress-activated protein kinases (SAPKs), the activation of caspases involved in apoptosis, an elevation of reactive oxygen species (ROS), and endoplasmic reticulum (ER) stress [9,10,11].

Microglial cells are immune cells in the central nervous system (CNS) that act as the first line of defense against infections and injuries. Their dysfunction has been linked to various neurodegenerative diseases, such as Alzheimer’s disease and Parkinson’s disease. At present, EMF-induced microglial cell toxicity and its regulatory mechanisms remain poorly understood. Natural substances that inhibit EMF-induced microglial cytotoxicity are also limited.

Polydeoxyribonucleotide (PDRN), a salmon sperm-derived DNA preparation with a 50–1500 base pairs [12], has demonstrated various biological benefits, including antioxidant, anti-inflammatory, wound healing, and regenerative properties [13,14]. Accumulating evidence indicates that PDRN and its metabolites, including adenosine, exert biological effects by interacting with and activating an adenosine A2A receptor (A2AR) signaling [15,16,17]. However, PDRN’s regulatory effect and mechanism on EMF-induced microglial cell toxicity have not been studied to date.

This study investigated whether exposure to EMFs at 3.5 GHz, potentially generated by smartphones working in 5G communication or cooking using microwave ovens, causes toxic effects on BV2 mouse microglial cells. Here, we hypothesize that acute exposure to 3.5 GHz electromagnetic fields (EMFs), which are commonly used in modern wireless telecommunication devices, triggers oxidative stress and apoptosis in BV2 microglial cells and that polydeoxyribonucleotide (PDRN) could inhibit such cytotoxicity through the modulation of ROS levels and stress-related protein kinases such as c-jun N-terminal protein kinase-1/2 (JNK-1/2), p38 mitogen-activated protein kinase (p38 MAPK), and caspase-9. The present study sought to investigate the mechanisms underlying EMF-induced microglial toxicity and to assess the therapeutic potential of PDRN.

Therefore, this study aimed to investigate whether PDRN can protect BV2 microglial cells from 3.5 GHz EMF-induced cytotoxicity and oxidative stress. Additionally, we explored whether this protective effect is mediated via the adenosine A2A receptor (A2AR) pathway using the selective A2AR antagonist ZM241385.

## 2. Materials and Methods

### 2.1. Materials

The primary antibodies, including eukaryotic initiation factor-2α (eIF-2α) (cat. no. 9722), phosphorylated (p)-extracellular signal-regulated protein kinase-1/2 (p-ERK-1/2) (T202/Y204) (cat. no. 9101), ERK-1/2 (cat. no. 9102), p-JNK-1/2 (T183/Y185) (cat. no. 9251), JNK-1/2 (cat. no. 9252), and p-p38 MAPK (T180/Y182) (cat. no. 9211) p38 MAPK (cat. no. 9212), p-MK2 (T334) (cat. no. 3007), and T-MK2 (cat. no. 3042), were obtained from Cell Signaling Technology, Inc. (Beverly, MA, USA). The primary antibody of p-eIF-2α (S51) (cat. no. ab32157) was purchased from Abcam (Cambridge, UK). Cell culture plates, including 6- or 24-well plates and a 100 mm dish, were procured from SPL Life Sciences (Pocheon, Gyeonggi-do, Republic of Korea). SP600125, SB203580, and PD98059 were purchased from Biomol (Plymouth, PA, USA). Dulbecco’s Modified Eagle Medium (DMEM) and fetal bovine serum (FBS) were obtained from Welgene (Daegu, Republic of Korea). The primary anti-procaspase-9 antibody (cat. no. ADI-AAM-139) was purchased from Enzo Life Sciences (New York, NY, USA).

Additionally, we sourced primary anti-β-actin antibody (cat. no. A5441), dichlorodihydrofluorescein–diacetate (DCFH-DA) (cat. no. D6883), N-acetyl cysteine (NAC) (cat. no. A9165), and other reagents from Sigma-Aldrich (St. Louis, MO, USA). PDRN (CELLVANE Inj.) was obtained from ZERONE CELLVANE, Inc. (Cheonan, Chungcheongnam-do, Republic of Korea).

### 2.2. Cell Culture

BV2 murine microglia cells were grown in DMEM supplemented with 10% heat-inactivated FBS, 2 mM glutamine, 100 U/mL penicillin, and 100 mg/mL streptomycin. BV2 cells were maintained at 37 °C in a humidified environment with 95% air and 5% CO_2_.

### 2.3. EMF Exposure Setup

**Figure 1:**
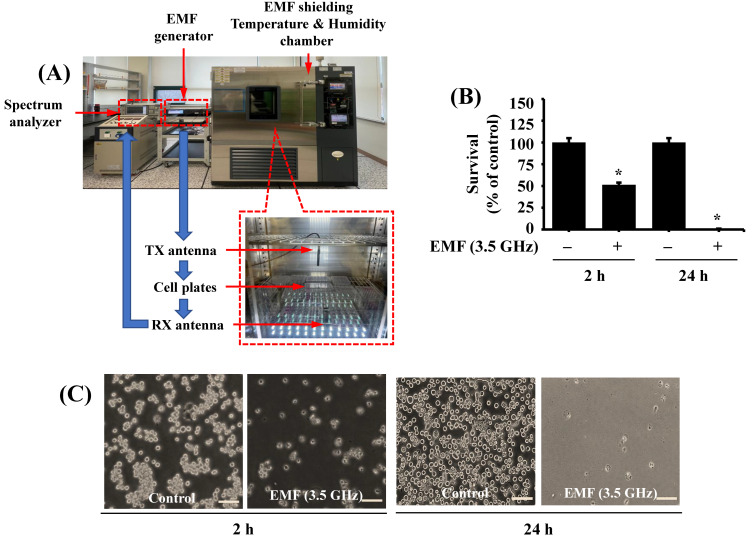
The EMF exposure system and the effect of 3.5 GHz EMF exposure on the growth of BV2 cells. (**A**) The specifically designed 3.5 GHz EMF exposure system and apparatus. (**B**) BV2 cells were exposed to either sham or 3.5 GHz EMFs for 2 or 24 h. The number of surviving cells was analyzed using cell count analysis. Data are presented as mean ± standard error (SE) from three independent experiments. * *p* = 0.001 indicates a significant difference from the control values (no 3.5 GHz EMF exposure) as determined by one-way ANOVA followed by Sidak’s post hoc test. (**C**) A representative image (Scale bar 100 µm) showing morphological changes in sham or 3.5 GHz EMF-exposed BV2 cells in (**B**).

We manufactured a specifically designed EMF exposure setup (Figure 1A). It is composed of an EMF generator for signal generation, a spectrum analyzer for signal frequency and strength observation, and a temperature and humidity chamber for EMF exposure environment building. Two omnidirectional antennas with a 3.1–5 GHz frequency range were connected to the EMF generator and spectrum analyzer to transmit and receive the signal individually. The cell plates were placed in the center of the chamber, and the signal transmission antenna (TX) was perpendicularly placed on the center of the cell plate to emit the radiation with the EMF generator output of 19 dBm at 3.5 GHz. Also, the receiving antenna (RX) was placed under the cell plate to measure the output from the EMF generator. The chamber is equipped with humidity and temperature systems and is set up to have 50% humidity and 37 °C, according to a previous report [18].

### 2.4. 3.5 GHz EMF Exposure to BV2 Cells

BV2 cells were seeded at a density of 0.12 × 10^6^ cells/0.5 mL/well in a 24-well plate or 0.6 × 10^6^ cells/2 mL/well in a 6-well plate the day before exposure. BV2 cells were then exposed to 3.5 GHz EMFs for 2 or 24 h. BV2 cells were pretreated without or with PDRN at the indicated doses for 1.5 h and further exposed without or with 3.5 GHz EMFs without or with PDRN or other drugs for the designated time points and doses. During EMF exposure, the experimental plates’ lids were intentionally left open to facilitate effective and uniform EMF penetration into cells. The plates were placed in the center of the exposure chamber below the antenna, and environmental parameters, such as humidity and temperature, were maintained at 37 °C and 50%, respectively, with tight regulation to minimize potential confounding caused by desiccation or thermal stress. Minimal open-lid time outside of EMF exposure was maintained with continuous monitoring for medium evaporation, which remained negligible after 2 h of exposure.

### 2.5. Cell Count Analysis

Control or EMF-exposed BV2 cells with or without PDRN or other drugs at doses tested were stained with 0.4% trypan blue dye (cat. no. 15250-061) (Gibco, Grand Island, NY, USA). Only cells with intact membranes can constructively exclude the dye, and then dead cells with damaged membranes become stained and counted using a light microscope. The cell count assay was performed in triplicate. Data are mean ± standard error (SE) of three independent experiments.

### 2.6. Measurement of DNA Fragmentation

DNA fragmentation assay was carried out according to the previously described method [19]. BV2 cells were seeded at a density of 0.6 × 10^6^ cells/2 mL/well in a 6-well plate overnight. Before EMF exposure, cells were pretreated without or with PDRN at the indicated doses for 1.5 h without 3.5 GHz EMF exposure. After 1.5 h pretreatment, cells were exposed to 3.5 GHz EMFs in the absence or presence of PDRN at the indicated doses for an additional 2 h. The conditioned cells were then harvested, washed, and lysed in a DNA isolation buffer [50 mM Tris (pH 8.0), 0.5% sarkosyl, 0.5 mg/mL proteinase K, and 1 mM EDTA] at 55 °C for 3 h, followed by the addition of RNase A (0.5 μg/mL) and incubation at 55 °C for 18 h. The cell lysates were further centrifuged at 13,000 rpm for 20 min. Genomic DNA was extracted with an equal volume of a neutral phenol–chloroform–isoamyl alcohol mixture (25:24:1) and analyzed by electrophoresis on a 1.8% agarose gel. The DNA was visualized and photographed under UV illumination after staining with ethidium bromide (0.1 µg/mL) by a gel documentation system (Gel Doc-XR, Bio-Rad, Hercules, CA, USA).

### 2.7. Preparation of Whole-Cell Lysates

After treatments, BV2 cells were washed twice with PBS supplemented with 1 mM sodium orthovanadate (Na_3_VO_4_) and 1 mM sodium fluoride (NaF) and subsequently exposed to cell lysis buffer [20 mM Tris-Cl (pH 7.5), 150 mM NaCl, 1 mM EDTA, 1 mM EGTA, 1% NP-40, 1% sodium deoxycholate, 2.5 mM sodium pyrophosphate, 1 mM β-glycerophosphate, 1 mM sodium vanadate, 1 mg/mL leupeptin, 1 mM phenylmethylsulfonyl fluoride]. The cells were then harvested and centrifuged for 15 min at 4 °C and 13,000× *g*. The supernatant was extracted, and protein concentrations were determined by bicinchoninic acid (BCA) protein assay (Pierce, Rockford, Tempe, AZ, USA) at 560 nm using a microplate reader (Bio-Rad Laboratories, Inc., Hercules, CA, USA).

### 2.8. Western Blot Analysis

Proteins (40 μg) were separated by SDS-PAGE (10%) and transferred onto nitrocellulose membranes (cat. no. IPVH00010) (Millipore, Bedford, MA, USA). The membranes were washed with a TBS solution (10 mM Tris, 150 mM NaCl) supplemented with 0.05% (*v*/*v*) Tween-20 [TBST], followed by blocking with a TBST solution containing 5% (*w*/*v*) non-fat dried milk. The membranes were incubated overnight at 4 °C with antibodies specific for procaspase 9 (1:2000), p-JNK-1/2 (1:2000), T-JNK-1/2 (1:2000), p-ERK-1/2 (1:2000), T-ERK-1/2 (1:2000), p-p38 MAPK (1:2000), T-p38 MAPK (1:2000), p-eIF-2α (1:2000), T-eIF-2α (1:2000), p-MK2 (1:2000), T-MK2 (1:2000), or actin (1:10,000). The membranes were then exposed to secondary antibodies coupled to horseradish peroxidase for 1 h and washed with a TBST solution. Immunoreactivities were detected by enhanced chemiluminescence (ECL) reagents. The level of actin protein was assessed by equal protein loading.

### 2.9. A2A Receptor Inhibition Using ZM241385

To investigate the involvement of the adenosine A2A receptor (A2AR) pathway in the protective effects of PDRN, BV2 cells were pretreated with ZM241385, a selective A2AR antagonist, at a concentration of 0.1, 0.5, and 1 μM. The inhibitor and PDRN were added to the culture medium for 1.5 h of EMF exposure. After pretreatment, cells were co-exposed to 3.5 GHz EMFs and treated with or without PDRN (100 μg/mL) and an inhibitor for 2 h. The experimental groups included EMFs alone, EMFs + PDRN, EMFs + ZM241385, and EMFs + ZM241385 + PDRN.

### 2.10. Measurement of Cellular ROS Levels

Levels of cellular ROS were detected with DCFH-DA, a fluorogenic dye, in BV2 cells exposed to 3.5 GHz EMFs or 0.2 mM H_2_O_2_, a known ROS inducer. DCFH-DA is a stable, nonfluorescent, nonpolar compound that permeates cell membranes. Once inside the cell, the acetyl groups are cleaved by cytosolic enzymes to form the polar nonfluorescent DCFH, rapidly oxidized to highly fluorescent DCF in the presence of ROS. Briefly, BV2 cells were plated at a density of 0.3 × 10^6^ cells/1 mL/well in a 12-well plate overnight. Cells were exposed to EMFs at 3.5 GHz or H_2_O_2_ at 0.2 mM for 1 or 2 h. In addition, cells were pretreated without or with PDRN at 100 µg/mL or NAC at 5 mM for 1.5 h without 3.5 GHz EMF exposure. After 1.5 h of pretreatment, cells were exposed to 3.5 GHz EMFs in the absence or presence of PDRN or NAC at the indicated dose for an additional 2 h. The conditioned cells were loaded with DCFH-DA to a final concentration of 10 μM for 30 min. BV2 cells were washed twice with PBS. Levels of cellular ROS were assessed using an inverted fluorescence microscope (Olympus Life Science, Shinjuku, Tokyo, Japan). Fluorescence intensity was quantified using ImageJ software1.52v, Java 1.8.0_112 (64-bit) , with the results expressed as percentage intensity relative to controls.

### 2.11. Statistical Analysis

Cell count analysis or Western blot for three independent experiments was measured in triplicate and repeated three times. The results were expressed as mean ± standard error (SE). One-way ANOVA followed by Sidak’s post hoc test was used to compare the significance of the difference. All significance testing was established on a *p*-value of <0.05.

## 3. Results

### 3.1. Exposure to 3.5 GHz EMFs for Either 2 or 24 h Significantly Inhibits the Growth of BV2 Microglial Cells

Using the specifically designed EMF exposure apparatus (Figure 1A), we initially investigated whether exposure to 3.5 GHz EMFs for 2 or 24 h influences the growth of BV2 cells using a cell count assay. Compared with the sham-exposed cells, the 2 or 24 h exposure to 3.5 GHz EMFs significantly reduced the growth of BV2 cells (Figure 1B). Microscopic observations further confirmed the capability of 3.5 GHz EMF exposure for 2 or 24 h to markedly inhibit the growth of BV2 cells. Due to the rapid and pronounced suppressive effects on BV2 cell growth, we chose the 2 h (and earlier than 2 h) exposure time of 3.5 GHz EMFs for further investigations.

### 3.2. Exposure to 3.5 GHz EMFs Leads to the Altered Phosphorylation and Expression Levels of JNK-1/2, ERK-1/2, p38 MAPK, eIF-2α, and Procaspase-9 in BV2 Murine Microglial Cells

**Figure 2:**
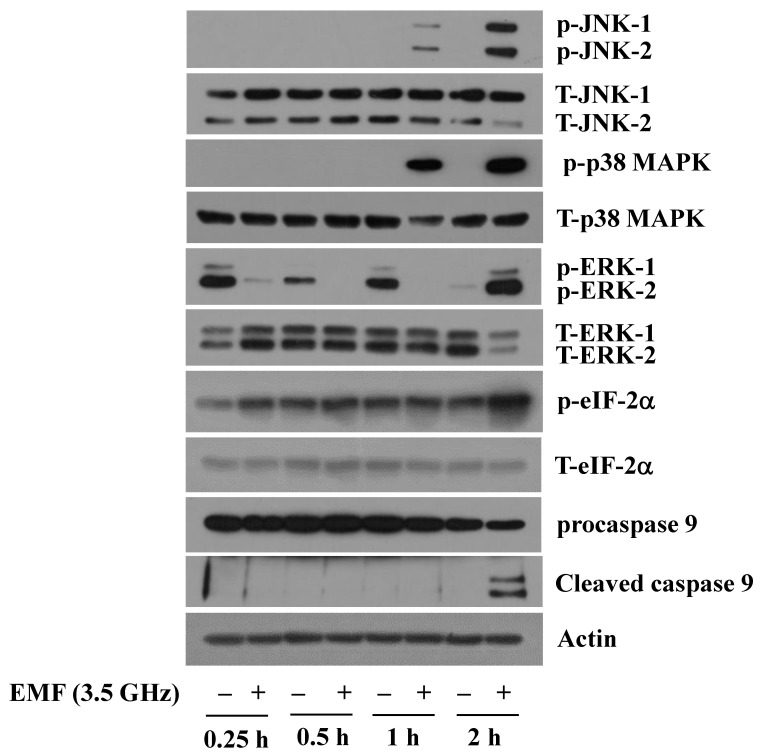
Effect of 3.5 GHz EMF exposure on the phosphorylation and expression of JNK-1/2, ERK-1/2, p38 MAPK, eIF-2α, and procaspase-9 in BV2 cells. BV2 cells were exposed without or with 3.5 GHz EMFs for the indicated times. At each time point, whole-cell lysates were prepared and analyzed by Western blotting with respective antibodies.

Next, to elucidate molecular and signaling mechanisms or factors responsible for 3.5 GHz EMF-mediated BV2 cytotoxicity, we examined the effect of exposure to 3.5 GHz EMFs on the phosphorylation and expression levels of growth and apoptosis-related proteins, including JNK-1/2, ERK-1/2, p38 MAPK, eIF-2α, and procaspase-9, in BV2 cells over time using Western blot analysis. As shown in Figure 2, compared with the sham-exposed cells, there was an increase in levels of phosphorylated JNK-1/2 and p38 MAPK in BV2 cells exposed to 3.5 GHz EMFs for 1 or 2 h. Shorter exposures to 3.5 GHz EMFs for 0.25 or 0.5 h did not affect the phosphorylation levels of JNK-1/2 and p38 MAPK in these cells. On the other hand, exposure to 3.5 GHz EMFs for 0.25, 0.5, or 1 h significantly reduced the phosphorylation levels of ERK-1/2 in BV2 cells but increased following 2 h exposure. In addition, compared with the sham-exposed cells, there was a notable increase in levels of phosphorylated eIF-2α in BV2 cells upon exposure to 3.5 GHz EMFs for 2 h. The respective total protein expression levels of JNK-1/2, p38 MAPK, ERK-1/2, and eIF-2α remained unchanged under these experimental conditions. Moreover, while exposure to 3.5 GHz EMFs for the times tested did not affect total protein expression levels of procaspase-9 in BV2 cells, 2 h of exposure to 3.5 GHz EMFs led to an elevation of cleaved (active) caspase-9 expression in these cells. Expression levels of the control actin protein remained constant under these experimental conditions. The time course experiment in Figure 2 was conducted to determine the time point of EMF exposure at which to conduct downstream experiments. All mechanistic experiments were conducted at the 2 h time point with *n* = 3 independent biological replicates based on peak phosphorylation at 2 h.

### 3.3. PDRN Blocks 3.5 GHz EMF-Induced Growth Inhibition and Apoptosis in BV2 Murine Microglial Cells

**Figure 3:**
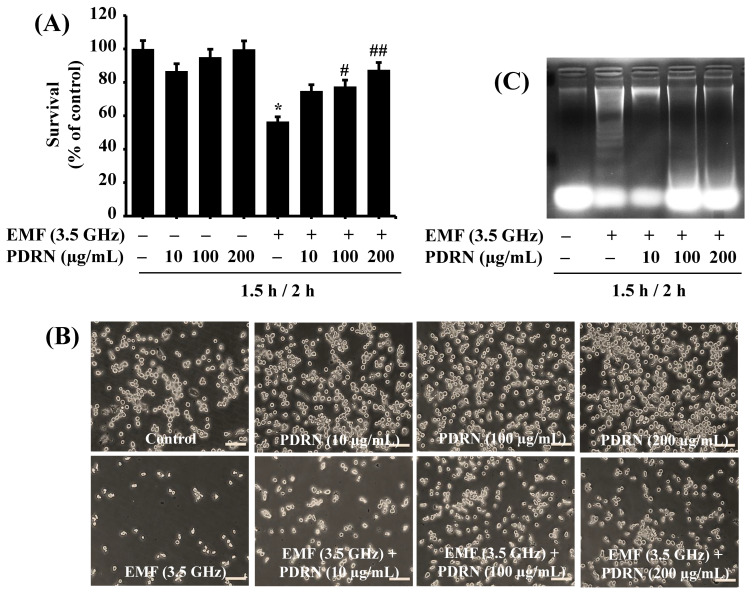
Effect of 3.5 GHz EMFs and PDRN on the growth and apoptosis of BV2 cells. (**A**) BV2 cells were pretreated without or with PDRN at the designated concentrations for 1.5 h and exposed to 3.5 GHz EMFs in the absence or presence of PDRN at the same doses for an additional 2 h. The number of surviving cells was analyzed using cell count analysis. Data are mean ± SE of three independent experiments. * *p* < 0.01 compared with the values of the control (no 3.5 GHz EMF exposure). ^#^ *p* < 0.02 and ^##^ *p* < 0.01 compared with the values of 3.5 GHz EMF exposure (with EMFs and PDRN) as determined by one-way ANOVA followed by Sidak’s post hoc test. (**B**) A representative image (Scale bar 100 µm) of morphological changes in sham or 3.5 GHz EMF-exposed BV2 cells in (**A**). (**C**) BV2 cells were pretreated without or with PDRN at the designated doses for 1.5 h and exposed to 3.5 GHz EMFs in the absence or presence of PDRN at the same doses for an additional 2 h. Extra-nuclear fragmented DNA from the conditioned cells was extracted and analyzed on a 1.8% agarose gel.

Next, we evaluated the effect of PDRN at different concentrations (10, 100, and 200 µg/mL) on 3.5 GHz EMF-mediated growth inhibition of BV2 cells. BV2 cells were pretreated with PDRN at the designated concentrations for 1.5 h, followed by exposure to 3.5 GHz EMFs for an additional 2 h, with or without the same concentrations of PDRN. As shown in Figure 3A, as anticipated, exposure to 3.5 GHz EMFs for 2 h significantly inhibited the growth of BV2 cells compared with the sham-exposed cells. Conversely, treatment with PDRN concentration dependently attenuated 3.5 GHz EMF-mediated growth inhibition of BV2 cells. Microscopic observations further demonstrated the ability of PDRN to dose dependently interfere with 3.5 GHz EMF-induced growth suppression of BV2 cells (Figure 3B). Subsequently, we assessed whether exposure to 3.5 GHz EMFs induces apoptosis in BV2 cells and whether PDRN blocks it using a genomic DNA fragmentation assay. As shown in Figure 3C, exposure to 3.5 GHz EMFs for 2 h induced nuclear DNA fragmentation in BV2 cells compared with the sham-exposed cells. However, (pre) treatment with PDRN at doses tested strongly abrogated it.

### 3.4. PDRN Inhibits 3.5 GHz EMF-Induced Generation of Cleaved Caspase-9, Phosphorylation of JNK-1/2, p38 MAPK, and ERK-1/2 in BV2 Murine Microglial Cells

**Figure 4:**
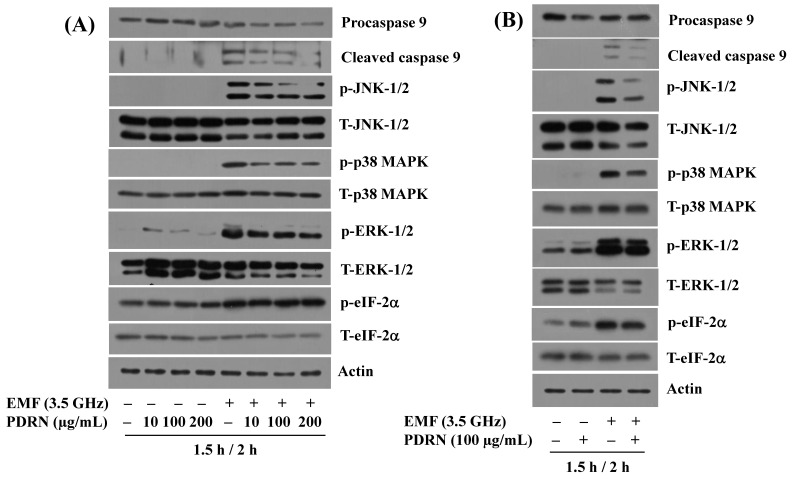
Effect of PDRN on the altered phosphorylation and expression of JNK-1/2, ERK-1/2, p38 MAPK, eIF-2α, and procaspase-9 induced by 3.5 GHz EMFs in BV2 cells. (**A**) BV2 cells were pretreated without or with PDRN at the indicated doses for 1.5 h and exposed to 3.5 GHz EMFs in the absence or presence of PDRN at the same concentrations for an additional 2 h. Whole-cell lysates from the conditioned cells were prepared and analyzed using Western blotting with antibodies. (**B**) BV2 cells were pretreated without or with PDRN at 100 µg/mL for 1.5 h and exposed to 3.5 GHz EMFs in the absence or presence of PDRN at the same concentrations for an additional 2 h. Whole-cell lysates from the conditioned cells were prepared and analyzed using Western blotting with antibodies.

Next, to understand how PDRN blocks 3.5 GHz EMF-induced growth inhibition and apoptosis in BV2 cells, we investigated whether PDRN at different concentrations (10, 100, and 200 µg/mL) modulates the altered phosphorylation and expression levels of JNK-1/2, p38 MAPK, ERK-1/2, eIF-2α, and procaspase-9 in BV2 cells in response to 2 h exposure to 3.5 GHz EMFs. As depicted in Figure 4, PDRN at concentrations tested significantly blocked 3.5 GHz EMF-induced generation of cleaved caspase-9 in BV2 cells. In addition, PDRN at doses applied substantially abolished the ability of 3.5 GHz EMFs to induce the phosphorylation levels of JNK-1/2, p38 MAPK, and ERK-1/2 without changing respective total protein expression levels in BV2 cells. However, PDRN did not affect the phosphorylation of eIF-2α induced by 3.5 GHz EMFs, nor did it influence the total protein expression levels of eIF-2α in BV2 cells. Further, we selected PDRN at 100 µg/mL; of note, as shown in Figure 4B, PDRN treatment partially blocks altered phosphorylation and expression levels of JNK-1/2, p38 MAPK, ERK-1/2, and procaspase-9 in BV2 cells in response to 2 h exposure to 3.5 GHz EMFs. Expression levels of the control actin protein remained constant under these experimental conditions. Extended triplicate results are provided in the supplementary materials (Figure S1).

### 3.5. Activation of JNK-1/2 and p38 MAPK Is Crucial for 3.5 GHz EMF-Induced Growth Inhibition of BV2 Murine Microglial Cells

**Figure 5:**
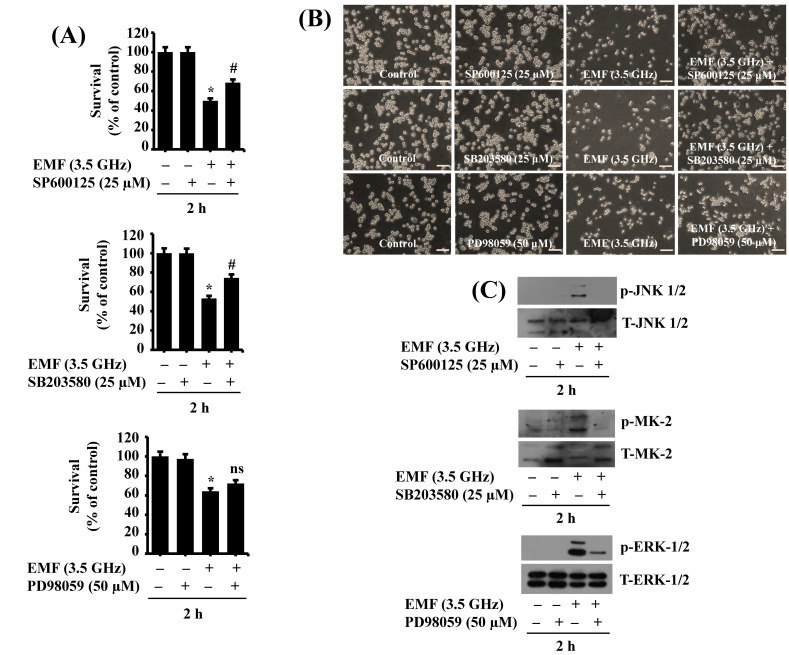
Effect of SP600125, SB203580, or PD98059 on 3.5 GHz EMF-induced growth suppression of BV2 cells and phosphorylation of JNK-1/2, p38 MAPK (MK2), or ERK-1/2 in BV2 cells. (**A**) BV2 cells were exposed to 3.5 GHz EMFs without or with SP600125 (25 μM), a JNK-1/2 inhibitor, SB203580 (25 μM), a p38 MAPK inhibitor, or PD98059 (50 μM), an ERK-1/2 inhibitor for 2 h. The number of surviving cells was analyzed using cell count analysis. Data represent the mean ± SE of three independent experiments. * *p* < 0.01 compared with the values of the control (no 3.5 GHz EMF exposure). ^#^ *p* < 0.01 compared with the values of 3.5 GHz EMF exposure (no drug), as determined by one-way ANOVA followed by Sidak’s post hoc test. (**B**) A representative image of morphological changes in the conditioned cells in (**A**). (**C**) BV2 cells were exposed to 3.5 GHz EMFs without or with SP600125 (25 μM), SB203580 (25 μM), or PD98059 (50 μM) for 2 h. Whole-cell lysates from the conditioned cells were prepared and analyzed using Western blotting with antibodies.

Next, we tested the role of phosphorylated (activated) JNK-1/2, p38 MAPK, and ERK-1/2 in 3.5 GHz EMF-induced growth inhibition of BV2 cells using pharmacological inhibition studies with SP600125, an inhibitor of JNK-1/2, SB203580, an inhibitor of p38 MAPK, or PD98059, an inhibitor of ERK-1/2. For this, BV2 cells were exposed to 3.5 GHz EMFs in the absence or presence of SP600125 (25 μM), SB203580 (25 μM), or PD98059 (50 μM) for 2 h, followed by measurement of the number of survived cells and the phosphorylation levels of JNK-1/2, p38 MAPK, and ERK-1/2 in these conditioned cells. As shown in Figure 5A, treatment with SP600125 or SB202380 significantly blocked 3.5 GHz EMF-induced growth inhibition of BV2 cells, whereas PD98059 treatment had little effect. Microscopic observations further confirmed the capability of SP600125 and SB203580, but not PD98059, to block 3.5 GHz EMF-induced growth inhibition of BV2 cells (Figure 5C). We next assured the efficacy of SP600125, SB203580, and PD98059 by measuring the phosphorylation levels of JNK-1/2, p38 MAPK, and ERK-1/2 induced by 3.5 GHz EMFs in BV2 cells, respectively. Given that SB203580 inhibits the activity of p38 MAPK without affecting the protein phosphorylation levels [20] and that p38 MAPK has several downstream effectors, including MAPK-activated protein kinase 2 (MAPKAP2, also known as MK2) [21], we herein assessed SB203580’s efficacy by alternatively measuring the phosphorylation levels of MK-2. As shown in Figure 5C, treatment with SP600125, SB203580, and PD98059 significantly suppressed 3.5 GHz EMF-induced phosphorylation of JNK-1/2, MK-2, and ERK-1/2 without affecting respective total protein expression levels in BV2 cells, respectively, pointing out each drug’s efficacy. Extended triplicate results are provided in the supplementary materials (Figure S2)

### 3.6. PDRN’s Blockage Effect on 3.5 GHz EMF-Induced Growth Inhibition of BV2 Murine Microglial Cells Is Dependent on the A2AR Pathway

**Figure 6:**
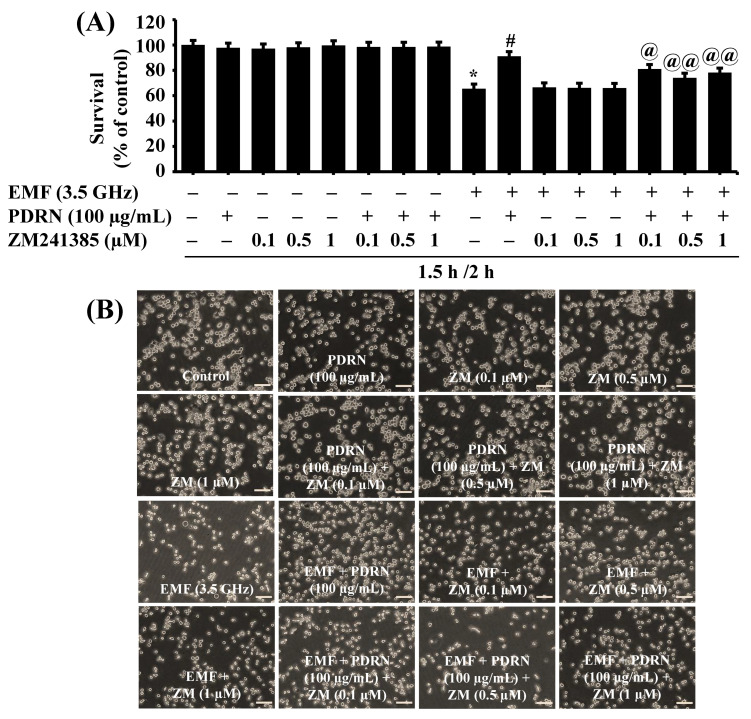
Effect of PDRN and ZM241385 on 3.5 GHz EMF-induced growth inhibition of BV2 cells. (**A**) BV2 cells were pretreated without or with PDRN or ZM241385, an A2AR antagonist, at the designated concentrations for 1.5 h and exposed to 3.5 GHz EMFs in the absence or presence of single or combined PDRN and ZM241385 at the same doses for an additional 2 h. The number of surviving cells was analyzed using cell count analysis. Data represent the mean ± SE of three independent experiments. * *p* < 0.01 compared with the values of control (no 3.5 GHz EMF exposure). ^#^ *p* < 0.01 compared with the values of 3.5 GHz EMF exposure (with EMF and PDRN). ^@^ *p* < 0.03 and ^@@^ *p* < 0.01 compared with the values of 3.5 GHz EMFs and PDRN (no drug), as determined by one-way ANOVA followed by Sidak’s post hoc test. (**B**) A representative image (Scale bar 100 µm) of morphological changes in the conditioned cells in (**A**).

PDRN is an A2AR agonist that exerts biological effects through the A2AR pathway. It promptly led us to test the role of A2AR in PDRN’s blockage effect on 3.5 GHz EMF-induced growth inhibition of BV2 cells using ZM241385, an A2AR antagonist. As shown in Figure 6A, compared with the sham-exposed cells, exposure to 3.5 GHz EMFs for 2 h led to a significant decrease in BV2 cell survival. On the other hand, PDRN at 100 μg/mL significantly abrogated 3.5 GHz EMF-induced growth inhibition of BV2 cells. In the absence of 3.5 GHz EMF exposure, either a single treatment with ZM241385 or a combined treatment with PDRN and ZM241385 at doses tested did not affect the growth of BV2 cells. A single treatment with ZM241385 at doses tested also did not influence 3.5 GHz EMF-induced growth inhibition of BV2 cells. However, ZM241385 at doses tested significantly attenuated PDRN’s protective effect on 3.5 GHz EMF-induced growth inhibition of BV2 cells. Microscopic observations further revealed ZM241385’s ability to vastly abrogate PDRN’s blockage effect on 3.5 GHz EMF-induced growth inhibition of BV2 cells (Figure 6B).

### 3.7. PDRN Significantly Inhibits 3.5 GHz EMF-Induced ROS Production and Growth Suppression of BV2 Murine Microglial Cells

**Figure 7:**
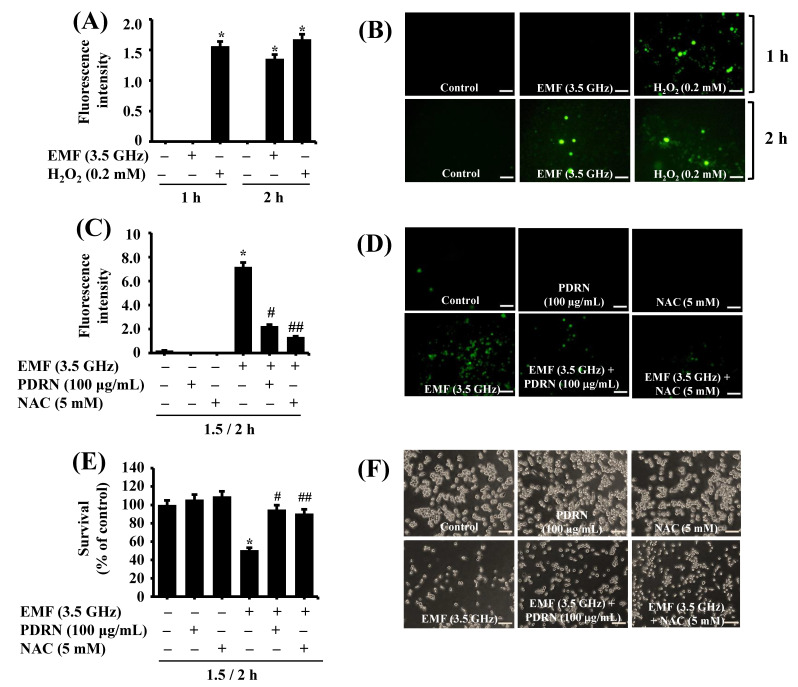
Effect of 3.5 GHz EMFs and PDRN on ROS generation in BV2 cells. (**A**) BV2 cells were exposed to 3.5 GHz EMFs or H_2_O_2_ for 1 or 2 h. At each time point, cells were loaded with DCFH-DA, and the conditioned cells’ DCF fluorescence intensity (ROS generation) was measured using fluorescence microscopy. Quantification was conducted by means of % intensity for each picture using ImageJ. DCF fluorescence is presented as mean ± standard deviation (SD) (*n* = 3) from the histogram statistics. ^*^ *p* < 0.01 vs. control (0 min) as determined by one-way ANOVA followed by Sidak’s post hoc test. (**B**) Intracellular ROS levels of the conditioned cells in (**A**) were measured by fluorescence microscopy (Scale bar 100 µm). (**C**) BV2 cells were pretreated without or with PDRN or NAC, an antioxidant, for 1.5 h and exposed to 3.5 GHz EMFs without or with PDRN or NAC at the indicated doses for an additional 2 h. The conditioned cells’ DCF fluorescence intensity (ROS generation) and quantification were measured using fluorescence microscopy and ImageJ, respectively. DCF fluorescence is presented as mean ± SD (*n* = 2) from the histogram statistics. * *p* < 0.01 compared with the values of the control (no 3.5 GHz EMF exposure). ^#^ *p* < 0.06 and ^##^ *p* < 0.02 compared with the values of 3.5 GHz EMF exposure (with EMFs, PDRN, and NAC) as determined by one-way ANOVA followed by Sidak’s post hoc test. (**D**) Intracellular ROS levels of the conditioned cells in (**C**) were measured by fluorescence microscopy (Scale bar 100 µm). (**E**) BV2 cells were pretreated without or with PDRN or NAC for 1.5 h and exposed to 3.5 GHz EMFs without or with PDRN or NAC at the designated dose for an additional 2 h. The number of surviving cells was analyzed using cell count analysis. Data represent the mean ± SE of three independent experiments. * *p* < 0.01 compared with the values of the control (no EMFs). ^#^ *p* < 0.02 and ^##^ *p* < 0.04 compared with the values of 3.5 GHz EMF exposure (with EMFs, PDRN, and NAC) as determined by one-way ANOVA followed by Sidak’s post hoc test. (**F**) A representative image (Scale bar 100 µm) of morphological changes in the conditioned cells in (**E**).

Next, we investigated whether exposure to 3.5 GHz EMFs elicits oxidative stress by measuring ROS levels in BV2 cells using confocal microscopy. This study used H_2_O_2,_ an ROS inducer [22], as a positive control. As shown in Figure 7A, exposure to H_2_O_2_ at 0.2 mM for 1 or 2 h led to an elevation of ROS in BV2 cells. Distinctly, while exposure to 3.5 GHz EMFs for 1 h did not elevate ROS levels in BV2 cells, 2 h of exposure increased ROS levels in these cells. Confocal microscopic observation further demonstrated the ability of 3.5 GHz EMFs or H_2_O_2_ exposure at times tested to induce green fluorescence (ROS production) in BV2 cells (Figure 7B). Next, we tested whether PDRN inhibits 3.5 GHz EMF-induced ROS production in BV2 cells. This study used NAC_,_ a known ROS scavenger [23], as a positive control. As shown in Figure 7C, NAC at 5 mM significantly blocked 3.5 GHz EMF-induced ROS production in BV2 cells. In addition, PDRN at 100 μg/mL significantly suppressed 3.5 GHz EMF-induced ROS production in BV2 cells. Fluorescence microscopic observation also exhibited the capability of PDRN or NAC to inhibit ROS generation induced by 3.5 GHz EMFs in BV2 cells (Figure 7D). Moreover, PDRN or NAC at doses tested significantly blocked 3.5 GHz EMF-induced growth inhibition of BV2 cells (Figure 7E). Microscopic observations further showed the capability of PDRN or NAC to vastly interfere with 3.5 GHz EMF-induced growth suppression of BV2 cells.

## 4. Discussion

The potential toxicity of EMFs at different frequencies from cell phones, microwaves, Wi-Fi, and other wireless devices on the brain has been a subject of ongoing research and debate. The 3.5 GHz EMF has been particularly notable due to its use in 5G networks and the potential for increased exposure as this technology is implemented. Chronic exposure to EMFs at 3.5 GHz is considered harmful to the brain, causing changes in neuronal activity, increased oxidative stress, and disruption of blood–brain barrier function [24,25]. Although the results have been mixed, animal studies have further shown cognitive impairment and altered brain function following long-term exposure to 2.45 GHz EMFs [26]. There is limited research explicitly addressing the toxic effect induced by acute 3.5 GHz EMFs in brain cells, including microglial cells. In this study, we investigated the toxic effects caused by acute exposure to 3.5 GHz EMFs on BV2 microglial cells as well as the PDRN regulation of these processes. Here, our findings suggest that ROS-mediated stress signaling plays a key role in EMF-induced cytotoxicity in BV2 microglial cells.

Through initial experiments, we have shown that 2 h of exposure to 3.5 GHz EMFs elicits BV2 cytotoxicity, characterized by its growth-suppressive and apoptosis-inducing effects on these cells. Exposure to EMFs can lead to various cellular changes in the brain, including increased oxidative stress, which may contribute to cellular dysfunction and many pathological processes in the CNS [27]. It is worth noting that exposure to 3.5 GHz EMFs causes high ROS generation and cellular damage in immortalized skin fibroblast cells and HaCaT keratinocytes [28]. Given that the 2 h of exposure to 3.5 GHz EMFs leads to an elevation of ROS in BV2 cells, but NAC, an antioxidant, significantly attenuates 3.5 GHz EMF-induced ROS production and growth inhibition of BV2 cells herein, it is plausible that ROS production appears crucial for 3.5 GHz EMF-induced BV2 cytotoxicity. PDRN is a natural substance with antioxidant properties [13]. PDRN regulation of 3.5 GHz EMF-induced ROS production and BV2 cytotoxicity is not fully elucidated. Of interest, the present study illustrates that PDRN at 100 μg/mL significantly inhibits 3.5 GHz EMF-induced ROS production and BV2 cytotoxicity, highlighting that PDRN’s protective effect on 3.5 GHz EMF-induced BV2 cytotoxicity is partially due to its antioxidant activity.

SAPKs, including JNK-1/2 and p38 MAPK, are a group of serine/threonine protein kinases that play a critical role in cellular responses to various stress stimuli, including EMF exposure [29]. Exposure to 2.45 GHz EMFs is reported to activate SAPKs in the brain, leading to the induction of stress response pathways and potentially contributing to neuronal damage and cell death [9]. There is evidence that JNK-1/2 and p38 MAPK are activated in response to EMF exposure, and their activations mediate the toxic effects of 918 MHz EMFs on astrocytes [30]. Supporting this, our findings also show the ability of 3.5 GHz EMF exposure to induce the phosphorylation (activation) of JNK-1/2 and p38 MAPK in BV2 cells. Considering the present findings that SP600125, a JNK-1/2 inhibitor, or SB203580, a p38 MAPK inhibitor, significantly abrogate 3.5 GHz EMF-induced BV2 cytotoxicity, it is likely that the activation of JNK-1/2 and p38 MAPK is crucial for the toxic effects of 3.5 GHz EMFs in BV2 cells. Previously, under inflammatory conditions, PDRN control of the activation of JNK-1/2 and p38 MAPK in different cell types has been reported [17]. However, PDRN regulation of 3.5 GHz EMF-induced activation of SAPKs in BV2 cells remains to be clarified. In the present study, PDRN at concentrations of 100 or 200 μg/mL vastly blocks 3.5 GHz EMF-induced activation of JNK-1/2 and p38 MAPK in BV2 cells. Thus, PDRN’s protective effect on 3.5 GHz EMF-induced BV2 cytotoxicity is further likely due to the inhibition of these SAPKs. Furthermore, ERK-1/2, a family of MAPKs, is a crucial signaling component in cell proliferation, differentiation, and survival [31]. It is shown that EMF exposure at 3.5 GHz can induce the activation of ERK-1/2 in microglial cells, leading to neuroinflammation and cellular dysfunction. Distinctly, in BV2 cells, EMF exposure at 3.5 GHz for 0.25, 0.5, or 1 h decreases ERK-1/2 phosphorylation, while exposure to 3.5 GHz EMFs at 2 h increases it. Given that PD98059, an ERK-1/2 inhibitor, does not alter 3.5 GHz EMF-induced BV2 cytotoxicity and PDRN at 100 or 200 μg/mL substantially inhibits ERK-1/2 activation in BV2 cells exposed to 3.5 GHz EMFs herein, it appears unlikely that the activation of ERK-1/2 is necessary for the toxic effects induced by 3.5 GHz EMFs in BV2 cells. Thus, PDRN’s protective effect on 3.5 GHz EMF-induced BV2 cytotoxicity does not seem to be due to the inhibition of ERK-1/2.

eIF-2α is crucial in regulating translation initiation [32]. The phosphorylation of eIF-2α, an inactive form of the protein [33], can result in the global inhibition of protein synthesis in cells [34], and its sustained phosphorylation often leads to cellular dysfunction and apoptosis [35,36]. In the current study, exposure to 3.5 GHz EMFs for 2 h leads to increased phosphorylation of eIF-2α in BV2 cells. However, considering that PDRN at doses and times tested has no effects on 3.5 GHz EMF-induced eIF-2α phosphorylation in BV2 cells herein, likely, 3.5 GHz EMF-induced growth suppression of BV2 cells is partially mediated through eIF-2α phosphorylation, and PDRN’s protective effect on 3.5 GHz EMF-induced BV2 cytotoxicity is unrelated to the control of eIF-2α phosphorylation.

Caspases are a family of cysteine proteases that play a critical role in regulating apoptosis [37]. Multiple lines of evidence show that caspases mediate apoptosis triggered by EMF-induced cellular stress [38]. Among the caspases, caspase-9 plays a vital role in initiating the apoptotic cascade in response to cellular stressors, like EMF exposure. Upon activation, caspase-9 can activate caspase-3, which cleaves various cellular proteins, leading to programmed cell death [39]. Given that 2 h of exposure to 3.5 GHz EMFs leads to the generation of cleaved caspase-9, an active form of the protein [40], in BV2 cells and PDRN dose dependently blocks it herein, it is likely that the activation of caspase-9 appears essential for the toxic effects induced by 3.5 GHz EMFs in BV2 cells, and PDRN’s protective effect on 3.5 GHz EMF-induced BV2 cytotoxicity is further partially mediated by inhibiting caspase-9.

PDRN and its metabolites, including adenosine, act as an agonist of A2AR [17]. Adenosine A2A receptors (A2ARs) are abundantly expressed in certain brain regions, particularly the striatum, under normal physiological conditions. In contrast, A2AR expression in glial cells, including microglia, is relatively low under basal conditions but is increased under pathological conditions such as neuroinflammation, neurodegeneration, and brain injury [41]. Reportedly, A2AR is expressed and can be activated by adenosine in BV2 cells [42]. The activation of A2AR signaling pathways may regulate neuroinflammatory responses in a context-dependent manner. For instance, A2AR activation was reported to promote anti-inflammatory effects and tissue repair in some settings, but in others, such as in activated microglia, A2AR activation can enhance the release of inflammatory cytokines [43]. With these two functions, the ability of PDRN, an A2AR agonist ZM241385, to block 3.5 GHz EMF-induced oxidative stress and apoptosis in BV2 cells indicates a context-dependent A2AR modulation protective function against acute EMF-induced stress states.

### Study Limitations and Future Directions

While this work introduces new mechanistic data on 3.5 GHz EMF-induced toxicity on microglial cells and the protective role of PDRN, some limitations should be acknowledged. BV2 cells, while used very widely as a model for microglia, are immortalized and may not fully reflect the phenotype and behaviors of primary microglia within tissues. Care should, therefore, be taken when extrapolating these results to more complex biological systems. For this, such findings are to be replicated in the future using primary microglial cultures or animal models in vivo, which will more accurately reflect physiological and pathological brain environments.

One of the major limitations of our current study is the use of acute, short-term EMF exposure, which will most likely not replicate real conditions, such as chronic or cyclical EMF exposure of cells and tissue. Several studies have noted that long-term or cyclical EMF exposure can lead to cumulative stress responses, oxidative disruption, and altered microglial activation patterns [44,45]. Such conditions can produce disparate molecular responses to acute exposure, especially under conditions of neuroinflammation and glial regulation.

While our original aim was to characterize the acute cytotoxic and oxidative actions of EMFs and the protective action of PDRN, we recognize the importance of investigating chronic and intermittent models of exposure. These models would likely be more representative of actual environmental EMF exposure and allow for the determination of the therapeutic window and effectiveness of interventions, like PDRN, over the long term. Future studies must, therefore, include longitudinal protocols that examine microglial response under these more representative conditions of exposure.

In addition, in our in vitro exposure setup, the culture plates were left open for EMF exposure to provide homogeneous and efficient field delivery at 3.5 GHz, as plastic lids can attenuate or reflect high-frequency signals. While this approach provides precise control of the exposure conditions, we acknowledge that it does not fully represent real-world scenarios in which biological tissues, such as the skin and skull, provide partial EMF attenuation. Therefore, future studies with tissue-equivalent barriers or in vivo models will be needed to further ascertain the contribution of physiological structures on EMF absorption and cell effects at more realistic exposure levels.

## 5. Conclusions

These results demonstrate firstly that acute exposure to 3.5 GHz EMFs causes BV2 cytotoxicity while PDRN blocks it, and PDRN’s blockage effect on BV2 cytotoxicity is mediated by regulating A2AR, ROS, JNK-1/2, p38 MAPK, and caspase-9. The present study advocates that PDRN can be utilized as a promising novel therapeutic agent against neurotoxic pathologies or diseases where microglial cell injury or damage is caused by acute exposure to 3.5 GHz EMFs. On the basis of our findings, we recommend a second set of experiments to explore the effect of chronic and intermittent EMF exposure on microglial cells. Such experiments would involve exposure paradigms, such as repeated daily cycles (e.g., 2 h on/2 h off) or constant low-dose EMFs over several days, along with dose-dependent studies of PDRN’s protective efficacy. Such investigations would give insight into whether PDRN still offers protection under conditions more representative of environmental EMF exposure and would also further elucidate its use as a neuroprotective drug.

## Data Availability

The data that support the findings of this study are available from the corresponding author upon reasonable request.

## Supplementary Materials

The following supporting information can be downloaded at: https://www.mdpi.com/article/10.3390/cimb47060386/s1.
