# Supplementary material for: Mitigation of 3.5 GHz Electromagnetic Field-Induced BV2 Microglial Cytotoxicity by Polydeoxyribonucleotide
Source: Curr Issues Mol Biol. 2025 May 22;47(6):386. doi: 10.3390/cimb47060386 (PMC12192555; doi:10.3390/cimb47060386)
Supplement: Supplementary file 1 [file cimb-47-00386-s001.zip › cimb-3584265-supplementary.pdf]

Supplementary Figure S1

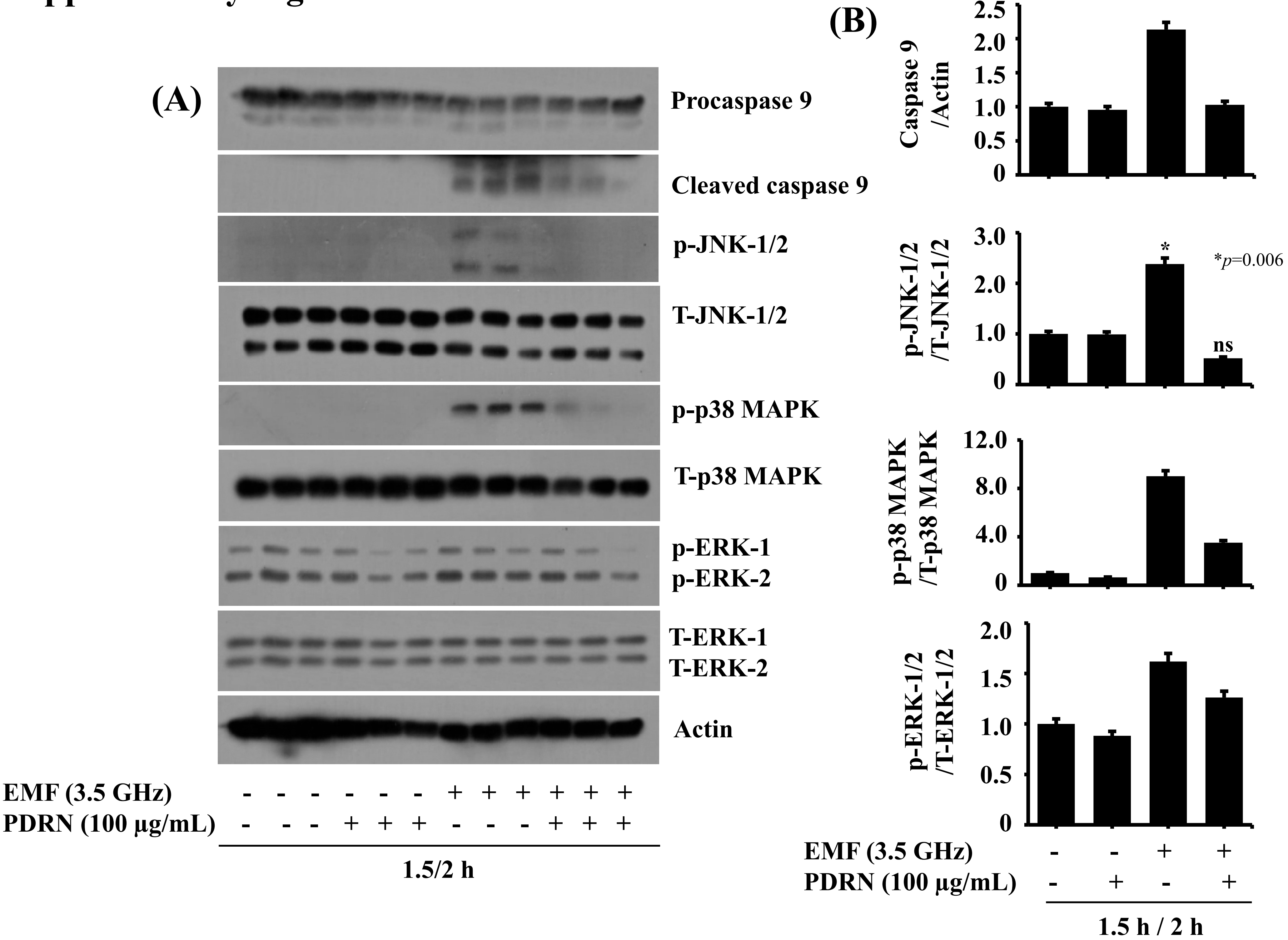

Supplementary Figure S2

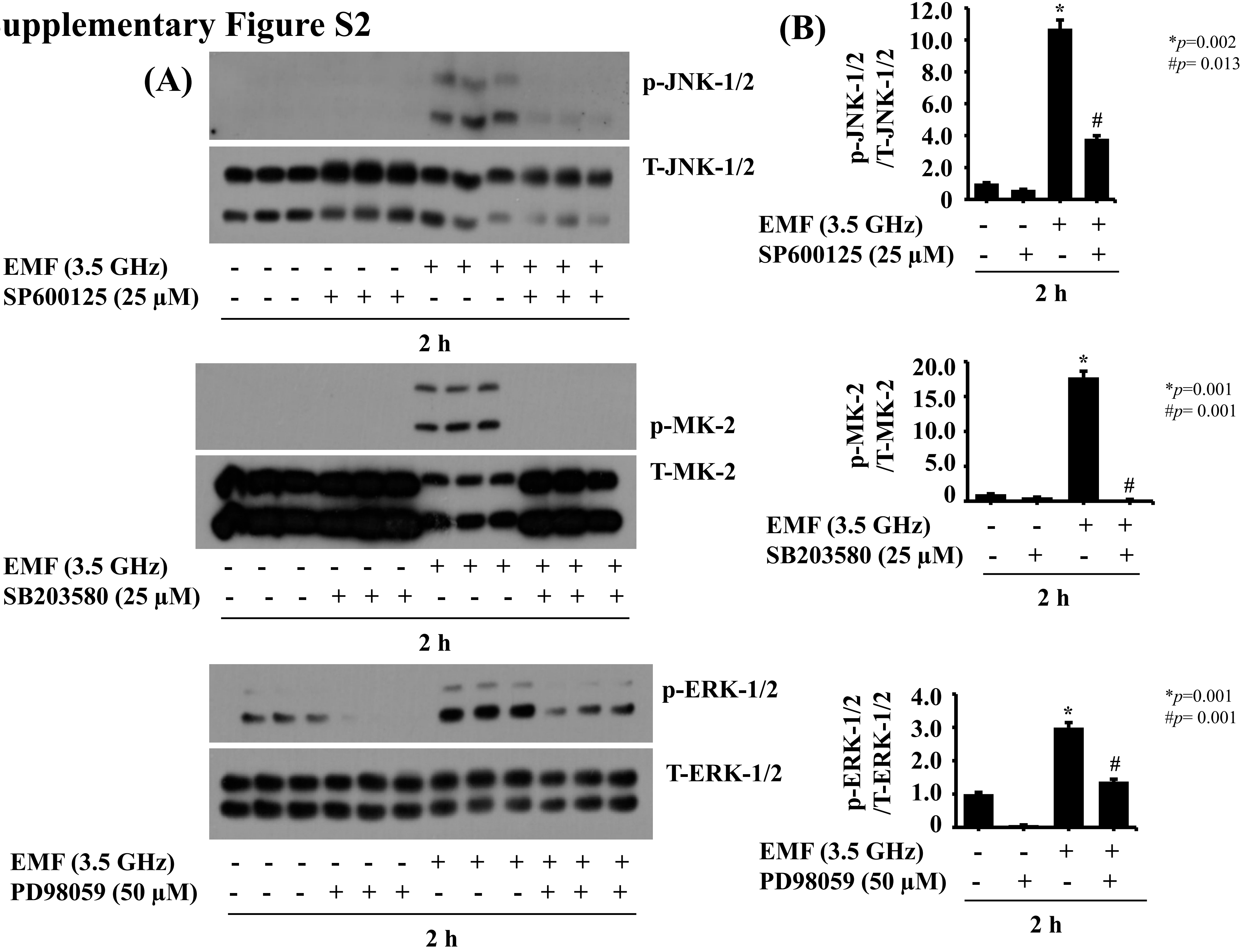

WB original blots- Supplementary Figure S1 A

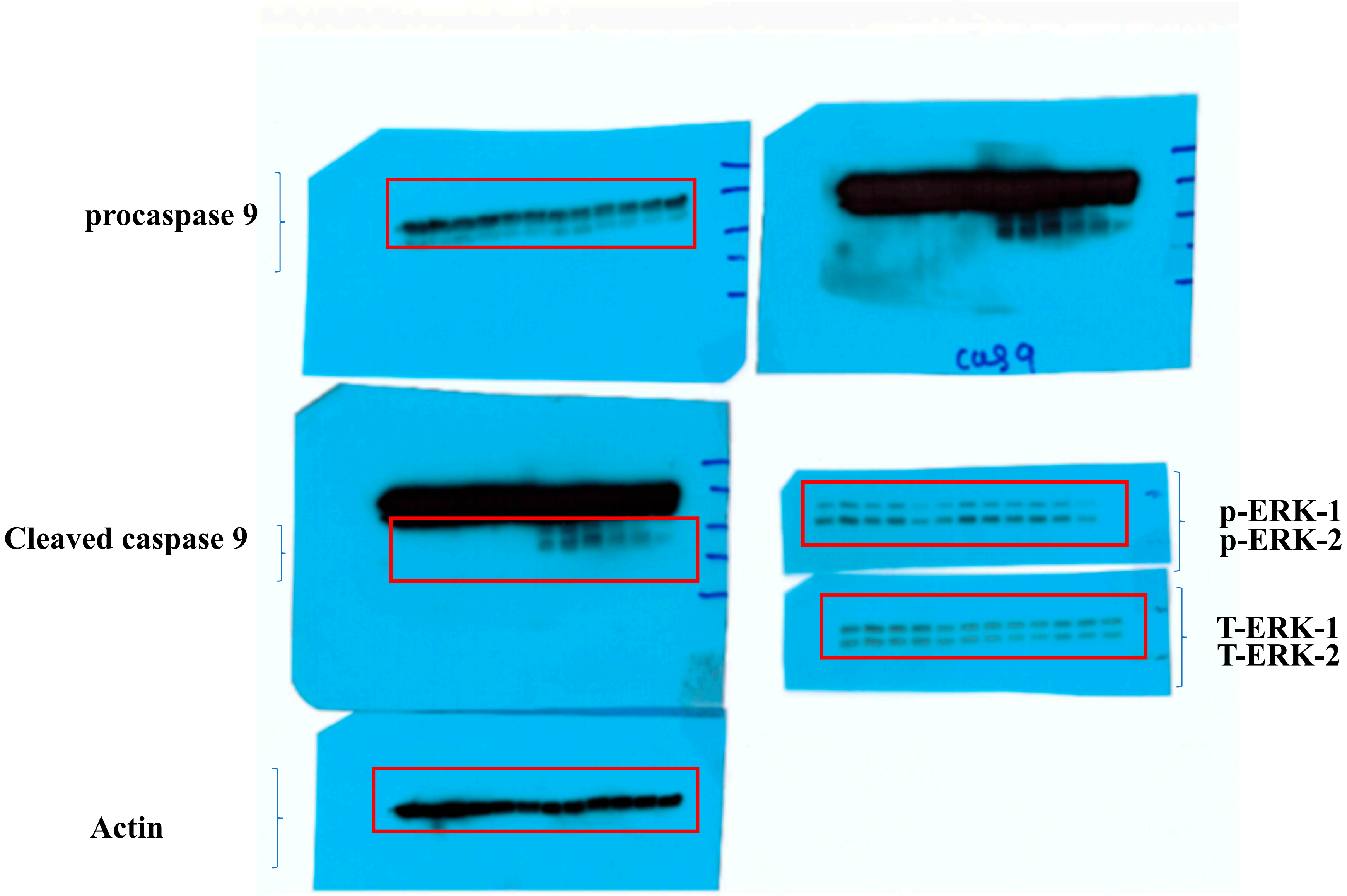

WB original blots- Supplementary Figure S1 A

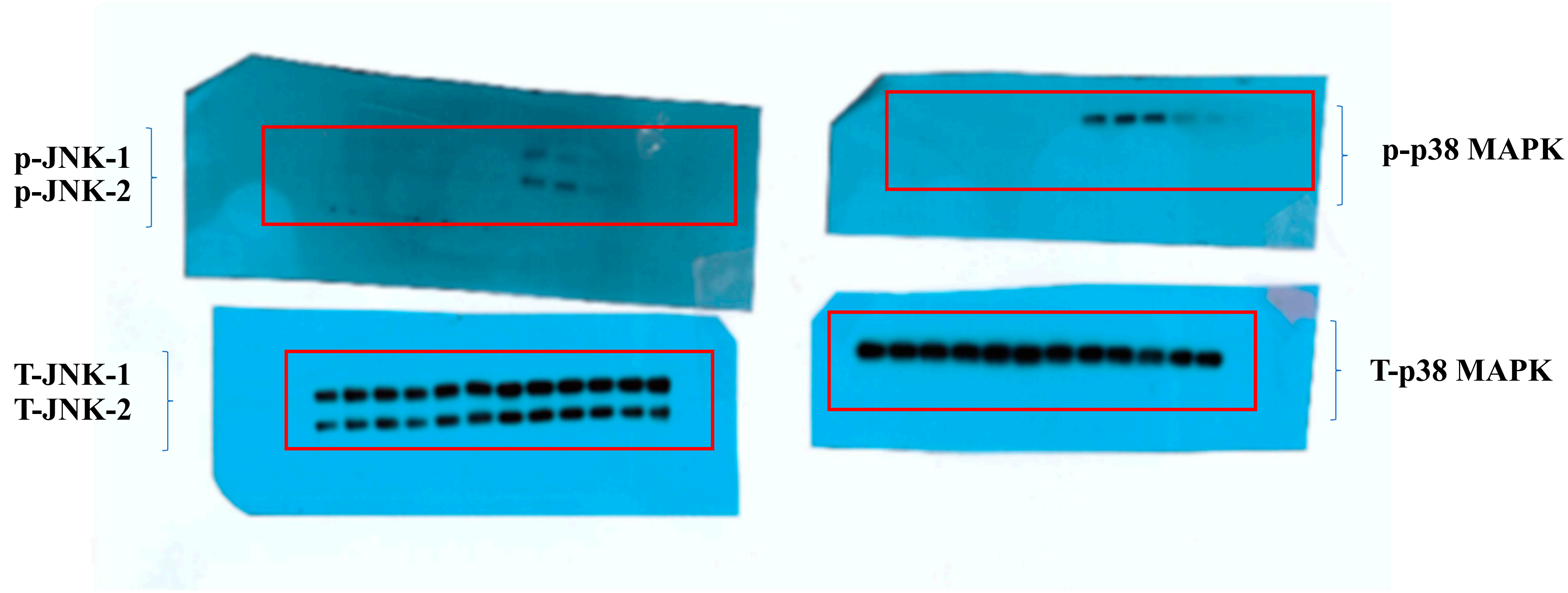

WB original blots- Supplementary Figure S2 A

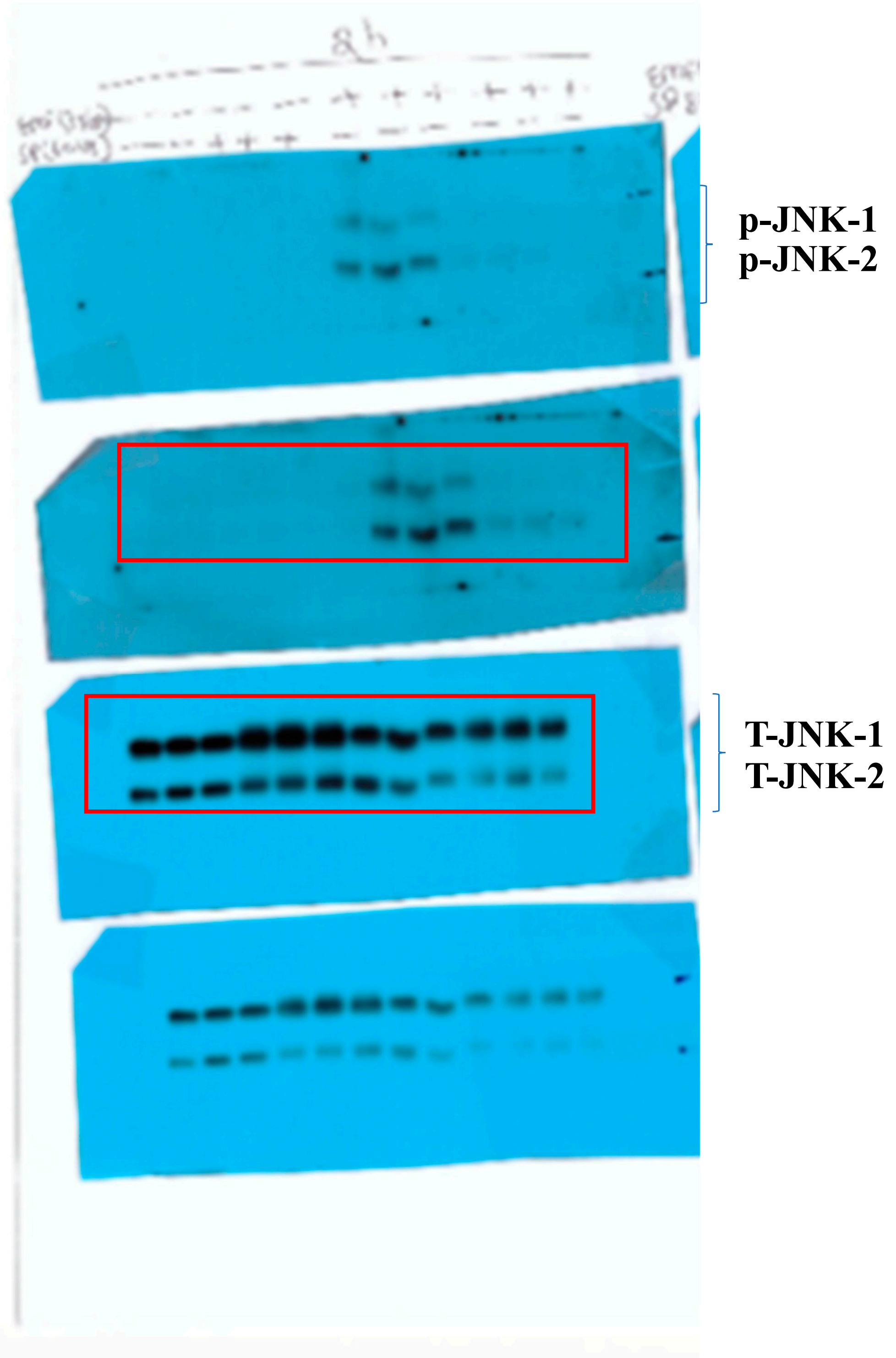

WB original blots- Supplementary Figure S2 A

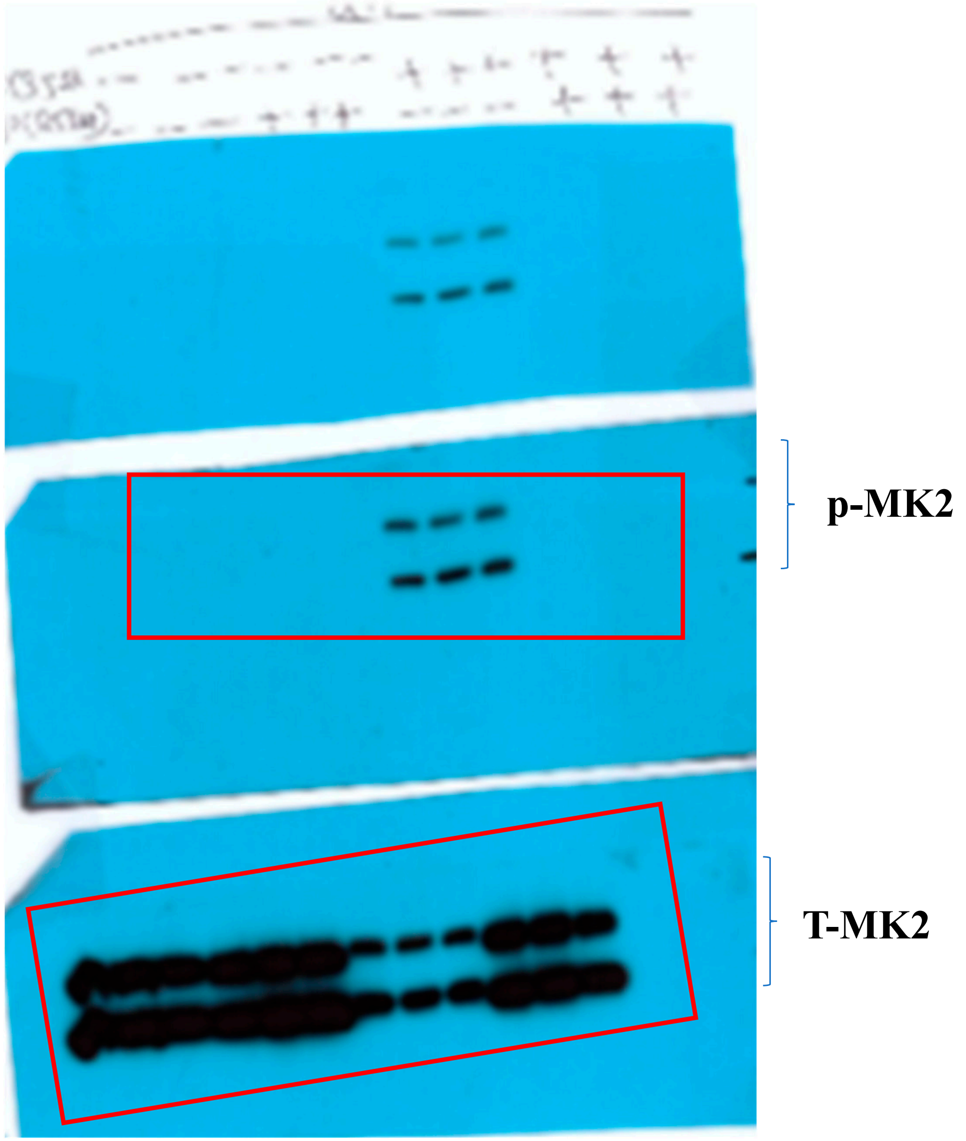

WB original blots- Supplementary Figure S2 A

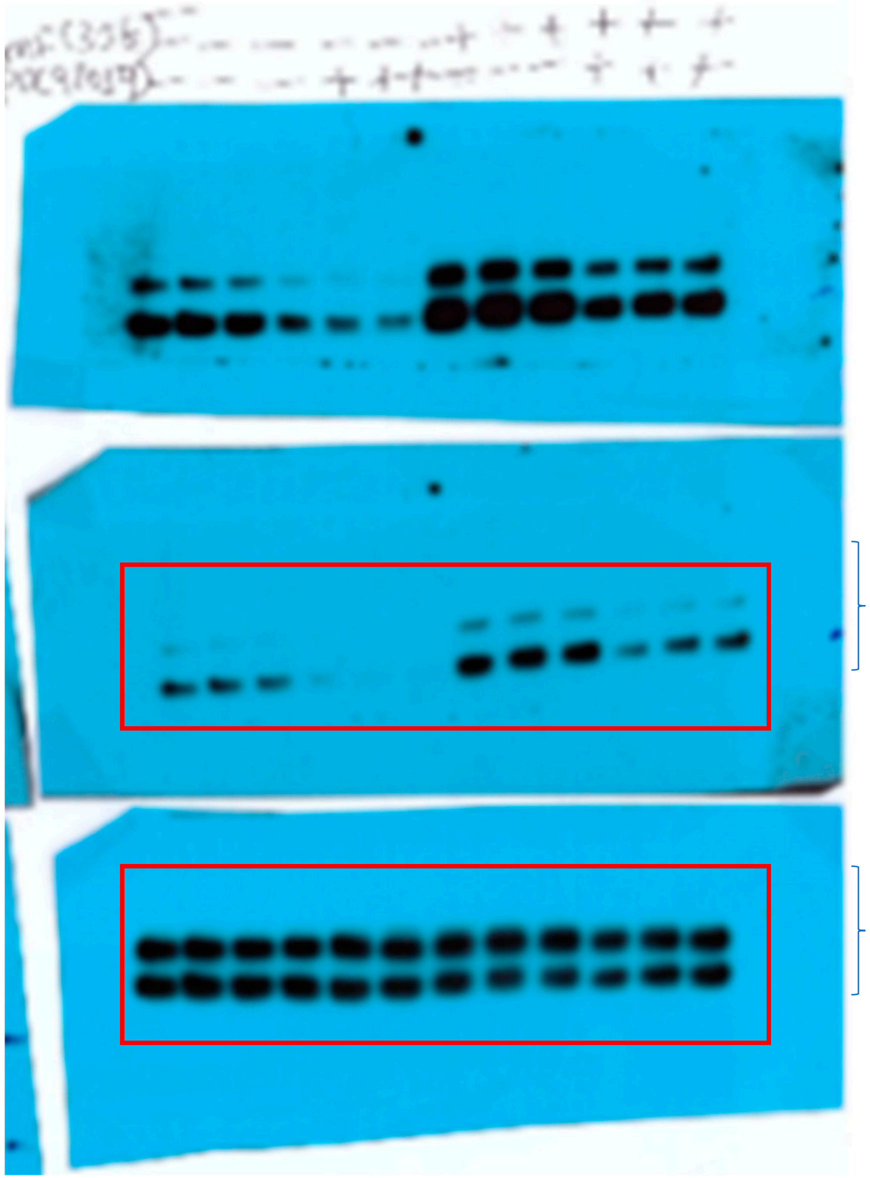

p-ERK-1
p-ERK-2

T-ERK-1
T-ERK-2
